# Mining flexible-receptor docking experiments to select promising protein receptor snapshots

**DOI:** 10.1186/1471-2164-11-S5-S6

**Published:** 2010-12-22

**Authors:** Karina S Machado, Ana T Winck, Duncan DA Ruiz, Osmar Norberto de Souza

**Affiliations:** 1 LABIO - Laboratório de Bioinformática, Modelagem e Simulação de Biossistemas. PPGCC, Faculdade de Informática, PUCRS, Av. Ipiranga, 6681 – Prédio 32, sala 602, 90619-900, Porto Alegre, RS, Brazil; 2 GPIN - Grupo de Pesquisa em Inteligência de Negócio. PPGCC, Faculdade de Informática, PUCRS, Av. Ipiranga, 6681 – Prédio 32, sala 628, 90619-900, Porto Alegre, RS, Brazil

## Abstract

**Background:**

Molecular docking simulation is the Rational Drug Design (RDD) step that investigates the affinity between protein receptors and ligands. Typically, molecular docking algorithms consider receptors as rigid bodies. Receptors are, however, intrinsically flexible in the cellular environment. The use of a time series of receptor conformations is an approach to explore its flexibility in molecular docking computer simulations, but it is extensively time-consuming. Hence, selection of the most promising conformations can accelerate docking experiments and, consequently, the RDD efforts.

**Results:**

We previously docked four ligands (NADH, TCL, PIF and ETH) to 3,100 conformations of the InhA receptor from *M. tuberculosis*. Based on the receptor residues-ligand distances we preprocessed all docking results to generate appropriate input to mine data. Data preprocessing was done by calculating the shortest interatomic distances between the ligand and the receptor’s residues for each docking result. They were the predictive attributes. The target attribute was the estimated free-energy of binding (FEB) value calculated by the AutodDock3.0.5 software. The mining inputs were submitted to the M5P model tree algorithm. It resulted in short and understandable trees. On the basis of the correlation values, for NADH, TCL and PIF we obtained more than 95% correlation while for ETH, only about 60%. Post processing the generated model trees for each of its linear models (LMs), we calculated the average FEB for their associated instances. From these values we considered a LM as representative if its average FEB was smaller than or equal the average FEB of the test set. The instances in the selected LMs were considered the most promising snapshots. It totalized 1,521, 1,780, 2,085 and 902 snapshots, for NADH, TCL, PIF and ETH respectively.

**Conclusions:**

By post processing the generated model trees we were able to propose a criterion of selection of linear models which, in turn, is capable of selecting a set of promising receptor conformations. As future work we intend to go further and use these results to elaborate a strategy to preprocess the receptors 3-D spatial conformation in order to predict FEB values. Besides, we intend to select other compounds, among the million catalogued, that may be promising as new drug candidates for our particular protein receptor target.

## Background

The pharmaceutical industry is under pressure to increase the rate with which it delivers new drugs to the market [1]. At present, the time to place a new drug into the market is between 10 to 15 years and the costs involved are estimated in 800 million dollars [2]. Due to these reasons there are current efforts towards changing these figures, for instance, by reducing the timeline and costs, and increasing the quality of the candidate drugs.

Advances in molecular biology and in computer modelling and simulation tools have had a direct impact in the drug discovery process, making viable the rational drug design (RDD) [3] approach. *In-silico* based RDD is a four-step cycle that combines structural information and computational efforts [4] based on a detailed understanding of the target protein (or receptor) and ligand interactions. In this sense, molecular docking algorithms are applied to evaluate and find the best ligand position and conformation inside the receptor binding site.

Nowadays, the majority of molecular docking algorithms consider only the ligand as flexible while the receptor remains rigid since it has far more atoms and consequently has a much greater number of degrees of freedom. It is computationally very expensive to consider the receptor flexibility [5] in molecular docking. Conversely, biological macromolecules like protein receptors are intrinsically flexible in their cellular environment. Therefore, it is very important to consider the receptor flexibility during molecular docking and, consequently, during RDD [6] because frequently the receptor can modify its shape upon ligand binding, moulding itself to be complementary to its ligand, increasing favourable contacts and reducing adverse interactions, thus minimizing the total free energy of binding (FEB) [7].

There are a number of alternative ways to incorporate at least part of the receptor flexibility. These have been reviewed by Teodoro and Kavraki [8], Totrov and Abagyan [9], Cozzini *et al*. [6], Huang and Zou [5], Wong [10], Alonso *et al*. [11] and Chandrika *et al*. [12]. Among these methods there are the approaches that consider one receptor conformation like the soft docking [13], the approach presented by Apostolakis *et al*. [14] and the methods that permit some mobility of the side-chains of the receptor binding site as devised by Leach [15] and the ones that use a rotameric library [16,17]. There are a large number of approaches that consider a set of receptor conformations. Some of these approaches combine the structures on a grid like the methods proposed by Knegtel *et al.*[18] and Österberg *et al.*[19]. Other approaches perform a series of docking experiments considering in each one a different receptor conformation. According to Teodoro and Kavraki [8] the first use of multiple structures derived from a molecular dynamics (MD) simulation was by Pang and Kozikowski [20]. Lin *et al*. [21,22] developed the relaxed complex scheme (RCS) to accommodate receptor flexibility in the search for correct receptor-ligand conformation. More recently, Amaro *et al*. [23] presented extensions of the RCS method which improves computational efficiency by reducing the receptor ensemble to a set of representative configurations.

In this work, we chose to model the explicit receptor flexibility by performing a series of molecular docking experiments considering in each one a different receptor snapshot derived from a MD simulation [24].

### Target receptor and ligands

Our target protein receptor is the InhA enzyme from *Mycobacterium tuberculosis* (MTB) [25]. This enzyme represents an important target to tuberculosis control [26]. Data from WHO [27] reports that about 9 million people will develop tuberculosis (TB) each year in the world and , at the same time, this disease will cause almost 2 million deaths. Furthermore, one third of the world’s population is infected with MTB [27,28]. More alarming is the growth of TB cases resistant to isoniazid and other anti-TB drugs [29]. In summary, these problems make it paramount to find alternative inhibitors for this enzyme.

**Figure 1:**
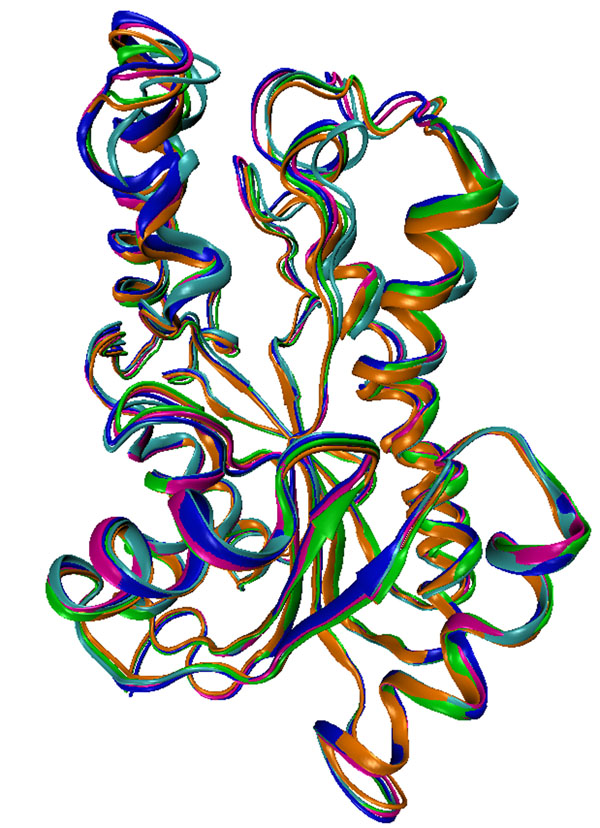
**Ribbon representations of 3-D conformations of the MTB’s InhA enzyme receptor.** The crystal structure (PDB ID: 1ENY) is coloured in orange. The other four conformations are averaged snapshots extracted from regions of a 3,100 ps MD simulation [31] trajectory of the InhA receptor. From 0.0 to 500 ps (cyan); 500 to1,000 ps (blue), 1,000 to 1,500 ps (magenta), and from 1,500 to 2,000 ps (green).

To illustrate the receptor flexibility, the 2.2 Å 3-D crystal structure (PDB ID: 1ENY) of InhA obtained from the Protein Data Bank (PDB) [30] can be viewed in Figure 1, together with four averaged conformations or snapshots extracted from different regions of the InhA 3,100 ps MD simulation trajectory [31]. Although simple, this example serves only to illustrates how flexible, by adopting different conformations, is the InhA receptor.

**Figure 2:**
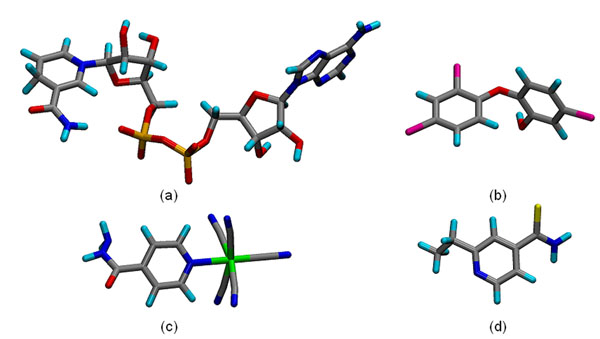
**Stick representation of the 3-D structures of the four ligands used in this work.** (a) NADH, (b) TCL, (c) PIF, and (d) ETH. The atoms are coloured by name: carbon (gray), nitrogen (blue), oxygen (red), hydrogen (cyan), phosphorus (orange), Iron (green), sulphur (yellow) and chlorine (magenta).

In this work we considered four different ligands, TCL [32], PIF [33], ETH [34] and NADH [25], which are summarized in Table 1. The ligands 3-D structures are illustrated in Figure 2. These structures were obtained either from the PDB [30] and ZINC [35], or generated by *ab initio* quantum mechanical methods [26].

**Table 1:** Names, abbreviations and the number of atoms of the ligands considered in this work.

Name	Abbreviation	Number of atoms
Nicotinamide adenine dinucleotide	NADH	52
Triclosan	TCL	18
Pentacyano(isoniazid)ferrate II	PIF	24
Ethionamide	ETH	13

The number of atoms comprehends the heavy and polar hydrogen atoms.

The 3,100 InhA receptor conformations (or snapshots) were obtained from a MD simulation trajectory as described in [31]. Considering this set of snapshots we performed molecular docking experiments [24] for each of the four ligands described. After the execution of over 3,000 docking experiments, for each ligand, as a result we have a large amount of data that need to be dissected to produce useful information about the receptor-ligands interactions. Then, we preprocessed all docking results and snapshots from the MD simulation and stored them into a proper repository developed and introduced in Winck et al, 2009 [36].

### Our contribution

In this article we propose a methodology to mine data from fully flexible-receptor molecular docking experiments, looking for receptor snapshots to which a particular ligand, amongst the four investigated here, binds more favourably.

We expect that our strategy can lead to the use of a significantly smaller number of snapshots in docking experiments with other ligands obtained from virtual libraries of small molecules. This advance is expected in the future to accelerate the molecular docking step of the drug discovery process while maintaining the level of detail with which we treat the receptor flexibility. To the best of our knowledge such an approach has never been explored. Amaro et al. [23] has also investigated snapshot selection from a MD trajectory. However in their approach they use QR factorization which measures the structural similarity between all pairs of Cα atoms among all the MD snapshots aligned.

To achieve our goal of snapshots selection we systematically preprocessed our molecular docking results and submitted them to the M5P model tree machine learning algorithm [37]. The model trees generated by this method were further post-processed. From these results we propose a criterion of selection of linear models (LMs) capable of picking out a set of conformations which are the most promising receptor conformations from the initial 3,100 snapshots for each ligand.

## Methods

### Full receptor flexibility from molecular dynamics simulation

The MD simulation of the InhA-NADH complex was performed for 3,100 ps as described in Schroeder et al. [31]. In this simulation the conformations were saved at every 0.5 ps which resulted in 6,200 (3,100 divided by 0.5) snapshots. However, for our work we took conformations at every 1.0 ps which resulted in 3,100 snapshots. This set of instantaneous receptor structures or snapshots was used to represent the full receptor explicit flexibility during the flexible-receptor docking procedure [24].

### Performing molecular docking experiments

For every ligand we submitted 3,100 docking experiments, with 10 runs each, using the scientific workflow proposed by Machado et al [24] using the simulated annealing (SA) protocol in the docking software Autodock3.0.5 [38]. In our laboratory we executed different docking experiments considering both flexible and rigid ligands. After evaluating the docking results, for the four ligands considered here, we did not observe differences that justified the use of flexible ligands in the docking experiments of the fully-flexible receptor. These findings were interesting because they allowed us to concentrate only in flexibility aspects of the receptor which is the most computationally demanding task in this type of receptor-ligand docking simulations. In summary, for the work presented here we used 3,100 snapshots of the MTB InhA enzyme receptor to represent its full explicit flexibility and all four ligands had their conformations kept rigid. With 10 runs per docking simulation we ended up with 31,000 results (receptor-ligand complexes and their estimated FEB) per experiment per ligand.

### The M5P model tree algorithm

As our entire attribute values in our mining input data are numeric and according to Han & Kamber [39] the most widely approach for numeric prediction is regression, we decided to explore this machine learning task.

Despite the lack of consensus in the data mining literature about the most understandable task result there is a reasonable agreement that representations such as decision trees and rule sets are better understood than black box representations such as Support Vector Machines or Neural Networks [40]. Decision trees have the advantage of being graphical representation of discovered knowledge and the tree hierarchical structure can point to information about the importance of the attributes used for prediction [40].

For prediction there are two main types of trees: regression trees and model trees. The main difference between these trees is in the content of the leaves. Each leaf in a regression tree stores a continuous-valued prediction that corresponds to the average value of the predicted attribute for the training tuples that get to the leaf. By contrast, in model trees, each leaf holds a regression model – a multivariate linear equation for the predicted value [39]. Owing to the multivariate linear equations generated by the model trees we decide to apply this algorithm to our input data. The model tree algorithm used in this work is the M5P [41] available in the WEKA package [42]. M5P handles tasks with very high dimensionality [41]. Indeed, our data mining input files have more than one hundred numeric attributes (shortest distances to residues) including the target attribute (the estimated FEB ). In our work, the M5P results can be especially useful because they could present an equation that properly weights the predictive attributes.

In model trees the input space is recursively partitioned until the data at the leaf nodes constitute subsets relatively homogeneous, so that a linear model can explain their variability [37]. Then, these linear models can be used to quantify the contribution of each predictive attribute to predict the target attribute.

### Application Tools

• AutoDock3.0.5 [38] is a suite of computer programs to perform automated molecular docking. It was developed to predict how small molecules bind to a receptor 3-D structure active site.

• AMBER 6.0 [43] is a suite of programs to energy-minimize and perform MD simulations of bio-molecules. It consists of a substructure database, a force field parameter file and also of a variety of utility programs. Ptraj, one of its modules, processes the trajectory files generated by the MD simulations.

• WEKA [42] is a collection of machine learning algorithms for data mining tasks. WEKA contains tools for data pre-processing and analyses by classification, regression, clustering, and association rules, as well as for their visualization.

• In-house developed Python scripts are used to pre- and post-process the data. For instance, to process the molecular docking outputs and to generate the proper mining inputs.

## Results

### The molecular docking outputs

**Figure 3:**
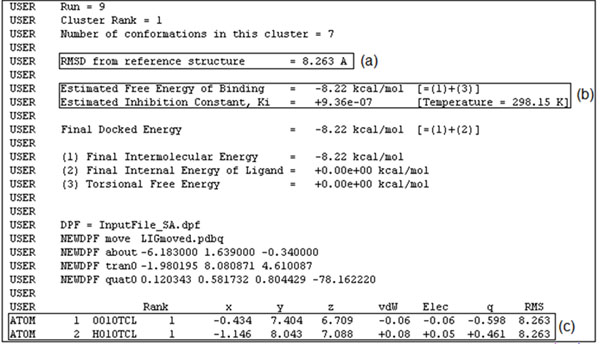
**Part of the AutoDock3.0.5 docking output file for a simulation of the InhA-TCL complex.** We specified 10 runs for each docking experiment. This figure shows the results of run 9, out of 10, with only the first and second atoms of the TCL ligand.

The output of a molecular docking simulation by AutoDock3.0.5 is a complex text file at the end of which the final docking results are summarized. An example of such a summary is shown in Figure 3. Each run result is mainly composed of three values highlighted by rectangular boxes in Figure 3: (a) Root Mean-Squared Deviation (RMSD), which indicates how distant the final ligand position is from its initial position; (b) the values of the estimated FEB and its corresponding inhibition constant (Ki); (c) the final 3-D coordinates of the ligand atoms.

Since we have 3,100 receptor snapshots and each docking simulation was set up with 10 runs, the maximum number of different ligand conformations inside the InhA active site is 31,000. However, during the docking simulations some of the runs either did not converge or had a positive FEB value. These data were considered outliers and were left out of the preprocessing of the docking results. Table 2 summarizes the docking results: for each ligand we have two lines; one with the results of all successful runs (maximum of 31,000) and another with only the best FEB of each docking simulation (maximum of 3,100). These are called the total number of valid results in the third column of Table 2.

**Table 2:** Results of the molecular docking simulations.

Experiments	Average FEB (Kcal/mol)	Number of valid results
NADH all runs	-9.2 ± 4.5	11,284
NADH best FEB	-12.9 ± 4.2	2,823
PIF all runs	-9.1 ± 1.6	30,420
PIF best FEB	-9.9 ± 0.6	3,042
TCL all runs	-8.2 ± 1.3	28,370
TCL best FEB	-8.9 ± 0.3	2,837
ETH all runs	-6.4 ± 0.3	30,430
ETH best FEB	-6.8 ± 0.3	3,043

The first column describes the ligands for each experiment and indicates the data size used to calculate the values in columns two and three. “all runs” refers to all docking runs. Its maximum value is 31,000. “best FEB” refers to the best estimated FEB for each set of simulations. Its maximum value is 3,100. The second column shows the average FEB (in Kcal/mol) and the third column the total number of valid results of each experiment.

As can be seen from Table 2, some docking simulations did not converge or had positive FEB values for PIF, TCL, and ETH, while many did not converge or had positive FEB for NADH, except for the NADH best FEB.

### Preprocessing inputs to data mining experiments

As exemplified at the end of the Background section, a virtual screening using a standard docking simulation of one receptor protein with 13 millions of compounds in the ZINC [35] library would take a long and an unacceptable time to complete. Adding full flexibility to the receptor, as we propose in this work, this time would be extraordinarily much bigger, turning virtual screening with this type of receptor flexibility model literally impossible and an efficient RDD process impractical. Our main interest is in developing ways to analyze and explore the data presented above in order to design some efficient strategy to speed up docking experiments with fully-flexible receptor. Our working hypothesis is: it is possible to perform *in silico* docking simulations for a given receptor-ligand pair, employing a reduced number of receptor snapshots, but still maintaining its full flexibility model. Consequently, we hope to answer the following question: “How to select a subset of snapshots, of the fully flexible-receptor model, which are most relevant to indicate whether a given ligand is a promising compound?”

The proposed approach to address our hypothesis and to start to answer the above question is to manage the large amount of data involved in docking simulations with a fully-flexible receptor model: the ligands, snapshots from MD simulations of the receptor and the results of their docking experiments. With this in mind, we will explore which data mining task can help us discover relationships in the receptor-ligands complexes. In this paper, we concentrate efforts on a regression mining algorithm to attempt to answer our working question. Our main contribution is in developing strategies focused on both preprocessing of data and post processing of mining results, aiming at obtaining predictive models to improve snapshot selection.

### Handling molecular docking simulation outputs and receptor’s snapshots

The FReDD repository [36] stores all features about the receptor snapshots, the ligands, and the docking simulation results used in this work. This repository allows easy retrieval of its information to produce comprehensive data to be mined.

The fully-flexible receptor model of the MTB InhA enzyme contains 3,100 snapshots, each with 4,008 atoms. This gives a total of 12,424,800 records for the atomic coordinates of the receptor.

The same calculation done for the InhA receptor is done for the four ligands used in this work. After the valid docking simulations (see Table 2) of the fully-flexible receptor with each ligand we end up with 568,768 records for NADH, 510,660 for TCL, 730,080 for PIF, and 395,590 for ETH (Table 3).

**Table 3:** Data size for preprocessing.

Ligand	Atoms	Number of valid results	Coordinates
NADH	52	11,284	586,768
PIF	24	30,420	730,080
TCL	18	28,370	510,660
ETH	13	30,430	395,590

Total		100,504	2,223,098

The first column shows the ligand names. The second, the total number of atoms of each ligand. The third, the total number of valid docking results, and the last column shows the total number of the ligands’s atomic coordinates stored in the FReDD [36] repository.

In summary, we have 12,424,800 and 2,223,098 records for the receptor and ligands, respectively.

### Generating the mining input data

**Figure 4:**
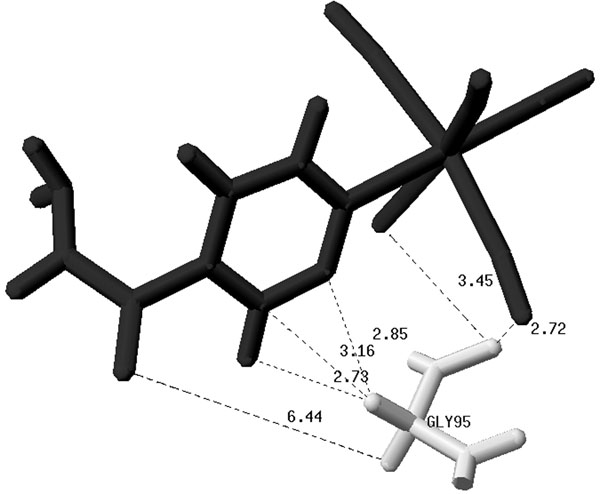
**Calculated inter-atomic distances between the ligand PIF and GLY95 residue of the InhA receptor.** The distances are in Å. For clarity only six out of 216 calculated distances for the GLY95-PIF pair are shown.

Predictive data mining algorithms concern with building predictive models that present the best relationships among a set of attributes, called predictive attributes, and a given attribute, called target attribute [44]. Regression tasks describe and distinguish the target attribute, which must be numeric, such that the resulting models can be used to predict the linear model (LM) to which the predictive attributes belong. In this work we use as predictive attribute the shortest distance between all atoms in the ligand and in the receptor’s residues, measured in ångströms (Å), and for each docking result. Figure 4 illustrates this concept. Its shows some distances between the PIF ligand and the receptor residue GLY95. From all of the calculated distances we consider only the shortest one. In this example, the shortest distance is 2.72 Å.

Therefore, for each snapshot there will be 268 such attributes which is equivalent to its number of residues. This procedure was repeated for the other three ligands. To generate the mining input data we needed to combine the 12,424,800 coordinate records of the receptor with the 2,223,098 records of all four ligands’ coordinates. It means that, applying Definition 1 below, which looks for the shortest distances between the receptor’s residues and ligand atoms, we have about 7 trillions of records for NADH, 6 trillion of records for TCL; 9 trillion of records for PIF, and 5 trillions of records for ETH.

Definition 1

The input data to mine for a specific ligand *l* ∈ {NADH, PIF, TCL, ETH} is a set *S* of tuples *<ssn, lc, SD, feb>, S⊆SSN ×LC ×Powerset(SD)×F,* such that:

• *ssn* is the receptor snapshot number,

• *lc* is the ligand *l* conformation,

• *feb* is the corresponding free energy of binding obtained from the docking experiment performed between snapshot *ssn* and ligand *lc,*

• *SD* is a list of 268 distance values *sd_i_*, *sd_i_* ∈ *SD,* between atoms from the snapshot *ssn* and ligand *lc,* and:

○ 1 ≤ *i* ≤ 268 corresponds to the *i^th^* protein receptor residue,

○ *ssn_a_ij_* is the *j^th^* atom of the *i^th^* protein receptor residue, and *ssn_a_ij_.x,ssn_a_ij_.y, ssn_a_ij_.z* are its corresponding spatial coordinates in *ssn*,

○ *lc_a_k_* is the *k^th^* atom of *lc,* and *lc_a_k_.x, lc_a_k_.y, lc_a_k_.z* are its corresponding spatial coordinates in *lc*,

○ *d_ijk_* = *SQRT((ssn_a_ij_.x - lc_a_k_.x)*^2^ + (*ssn_a_ij_.y - lc_a_k_.y*)^2^ + (*ssn_a_ij_.z - lc_a_k_.z*)^2^) is the Euclidian distance between *ssn_a_ij_* and *lc_a_k_*,

○ *sd_i_* = *MIN(d_ijk_)*, where *i* is the *i^th^* protein residue, *ij* corresponds to the *j^th^* atom of the *i^th^* protein receptor residue, and *k* corresponds to the *k^th^* atom of *lc* ligand.

Having these results, the next step is to arrange them into an appropriate input file or table which is composed of 271 attributes. Table 4 describes part of such an input file for PIF. The first column contains the number of the receptor snapshot in the same order as it appears in the MD simulation trajectory. The second column contains the ligand conformation for each of the 10 runs in a docking simulation. The next 268 columns hold the shortest distance found for each receptor residue, and, finally, the last column contains the estimated FEB value which is our target attribute.

**Table 4:** Example of part of a mining input data file for PIF. See text for details.

Receptor Snapshot	Ligand Conf.	…	Res. 13	…	Res. 268	FEB (Kcal/mol)
1	1	…	1.95	…	20.38	-9.94
…	…	…	…	…	…	…
94	461	…	3.18	…	24.60	-10.91
…	…	…	…	…	…	…
3,100	30,420	…	4.21	…	18.99	-9.61

### Improving the input file

The largest distance value that allows a biologically meaningful contact between receptor and ligand atoms is 4.0 Å [45,46]. Hence, distances higher than 4.0 Å means that the corresponding receptor residue does not establish a direct contact with some atom of the ligand.

To improve the quality of the models, we removed all attributes (residues) to which the shortest distance to any ligand is bigger than 5.0 Å (recall Definition 1): *S’ ⊆S* is a set of tuples *s’ = <ssn, lc, SD’, feb>,* where

• *ssn, lc* and *feb* have the same meaning as in Definition 1,

• *SD’* is a list of *n* values, *n ≤* 268, and

• *∀sd_i_ ∈ SD, sd_i_ ∈ SD’ ↔ ∃s_m_ ∈ S | s_m_ = <ssn_m_, lc_m_, SD_m_, feb_m_> ^ sd_j_ ∈ SD_m_ ^i = j ^ sd_j_*≤ 5.0 Å.

We chose 5.0 Å in order to consider a 1.0 Å margin of risk. After this feature selection, instead of the 268 original residues for each ligand, we ended up with 106, 122, 121 and 128 receptor residues, respectively, for NADH, PIF, TCL and ETH.

### Applying the M5P model tree algorithm

To obtain linear models to select the most promising snapshots having the described input data we need to apply a data mining algorithm that would be capable to predict the FEB value based on the shortest receptor residues-ligand distances, establishing a relationship between them. We choose the M5P model tree algorithm [41].

We performed one experiment applying M5P for each ligand, based on the instances in the preprocessed input files. In addition, we removed their first two columns. Among the parameters available in M5P, we concentrated in calibrating parameters related to legibility and accuracy of the generated model trees. The M5P minimum number of instances parameter is related to the size of the resulting model tree and the number of linear models (LM) generated by the algorithm. Accordingly, we set this parameter to 1,000 for all mining experiments.

**Figure 5:**
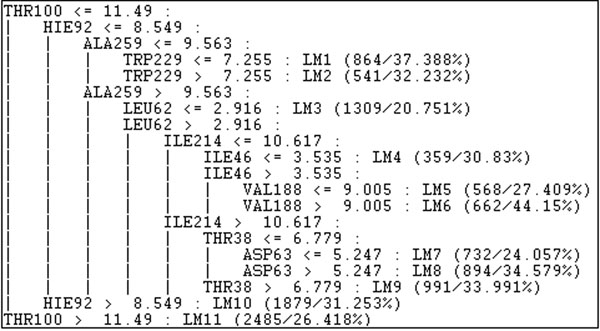
**Model tree of NADH in a M5P algorithm output using WEKA.** The model tree of NADH has a total of 10 nodes with 11 LMs.

To exemplify the result of M5P we present in Figure 5 the model tree for the NADH ligand that has 10 nodes with 11 LMs. This tree is in the format of the M5P algorithm output using WEKA. Each node corresponds to a receptor residue and the decisions in the tree are related to the residues distances to the NADH ligand.

Each leaf node is a LM in the form described by Equation (1) that corresponds to the LM1 in the NADH model tree described in Figure 5.

(1) **FEB =** 0.0003*PHE22 - 0.0001*THR38 + 0.005*ILE46 + 0.0012*LEU62 + 0.0041*ASP63 + 0.0001*VAL91 - 0.0002*HIE92 + 0.0006*THR100 - 0.0007*GLY101 + 0.0013*LEU167 + 0.0061*VAL188 - 0.0002*GLY191 + 0.0039*ILE193 + 0.0036*ILE214 + 0.0122*TRP229 - 0.0051*ALA259 **- 10.5158**

The results for all ligands are summarized in Table 5. In this table we show, for each ligand, the number of instances and the number of attributes submitted to the algorithm; the number of nodes in the tree and its depth; the total number of LMs produced followed by correlation. On the basis of the correlation values, we were able to generate very satisfactory models. These models produced short and understandable trees. We can see that for NADH, TCL and PIF we obtained more than 95% correlation. ETH, however, showed the worst correlation, which was about 60%. The reason why ETH correlation is so low is because it is pro-drug. It binds to the InhA receptor protein active site as an adduct with NADH (ETH-NADH). In the docking experiments explored here we considered only the ETH molecule. Alone, ETH explored a larger area of the receptor active site that is actually not available to it in its inhibitory form (ETH-NADH adduct). Despite the large difference in Correlation as compared to the other three ligands, its 60% correlation is a very satisfactory value. We know from visual inspection that ETH alone preferred to bind to or near the active site region of InhA where it binds as ETH-NADH inhibitory adduct.

**Table 5:** Results of the mining experiments with M5P.

	NADH	TCL	PIF	ETH
Instances	11,284	28,346	30,142	30,430
Attributes	107	122	123	129
Nodes	10	22	18	18
Depth	7	10	7	7
LM	11	23	19	19
Correlation	0.9510	0.9717	0.9689	0.6022

The second, third, fourth and fifth columns describe the results for the NADH, TCL, PIF and ETH respectively. The first line describes the total number of considered instances of each ligand. The other lines show characteristics of the model trees.

Despite the overall high quality of the correlation values, we still need to apply the model trees on a test set to evaluate whether they can really help us to achieve proper snapshot selections. In face of this, from now on we focus our attention in formulating a strategy to identify the LMs capable of predicting, and thus selecting, the most promising receptor snapshots.

### Post processing and evaluating the models results

As our objective is to select snapshots we need to establish a selection criterion of LMs. That is, we have to identify which are the best LMs in order to traverse the tree and select the snapshots that belong to the selected LMs. Therefore, the snapshots in the instances classified in the selected LMs indicate the most promising receptor snapshots to be considered in future docking simulations. That evaluation phase is composed of three steps as follows:

1. We traverse the produced trees with our test set to identify which instances belong to which LM.

2. We carefully establish a criterion to select representative LMs;

3. We evaluate whether the selected snapshots are indeed promising.

### Selecting representative LMs

Each ligand test set is composed of the receptor snapshot collection considering only the docking results with best FEBs (Lines 2, 4, 6, and 8 of Table 2). We chose to have one test set for each ligand because, with exception of NADH, the other three ligands are chemically distinct, but with similar biological role: they are inhibitors of the InhA receptor function.

We started by implementing Python scripts to map the instances according to the LMs of each model tree. These scripts verify to which LMs belongs each one of the instances of the test set. Having completed this mapping, we are ready to indicate which LMs are representatives, and thus use them to select the snapshots. We have as a premise that good snapshots are those ones that present low FEB values. However, such values vary from one snapshot to another. We take as a starting point the average FEB (column 2 of Table 2) from each test set. For each LM, we calculate the average FEB for their associated instances. From these values we set up the following LM selection strategy: **a given LM is considered representative if its average FEB is smaller (more negative) than or equal to the average FEB of the test set.**

To exemplify our methodology of LMs selection we consider the results for the PIF ligand. Table 6 describes in the columns 1 and 4 the LMs; columns 2 and 5 the total number of instances for each LM while columns 3 and 6 contain the average FEB values for each LM. Based on our strategy, as the average FEB of the PIF test set is -9.9 Kcal/mol (Table 2) the correspond LMs selected for this ligand are LM1, LM2, LM3, LM5 and LM7 (highlighted in bold in Table 6).

**Table 6:** Analysis of the LMs generated for the PIF ligand.

LM	Total of instances	Average FEB (kcal/mol)	LM	Total of instances	Average FEB (kcal/mol)
**LM1**	**1,776**	**-9.98**	LM11	250	-9,65
**LM2**	**91**	**-10.28**	LM12	131	-9,57
**LM3**	**48**	**-10.15**	LM13	26	-9,76
LM4	96	-9.74	LM14	14	-9,32
**LM5**	**65**	**-9.93**	LM15	3	-8,98
LM6	178	-9.79	LM16	11	-4,88
**LM7**	**105**	**-9.90**	LM17	6	-4,78
LM8	38	-9.77	LM18	0	-
LM9	60	-9.71	LM19	2	-4,44
LM10	142	-9.53			

Tables 7, 8 and 9 have the same format of Table 6 and describe all the results obtained for all the other three ligands: NADH, TCL and ETH. For example, for NADH (Table 7) the average FEB is -12.90 kcal/mol. We can observe that only LM11 has an average FEB smaller than this value. Consequently, LM11 is selected for NADH. All the selected LMs for each ligand are highlighted in the tables.

**Table 7:** Analysis of the LMs generated for the NADH ligand.

LM	Total of instances	Average FEB (kcal/mol)	LM	Total of instances	Average FEB (kcal/mol)
LM1	257	-10.67	LM7	53	-8.06
LM2	153	-8.43	LM8	141	-7.71
LM3	255	-9.39	LM9	87	-6.84
LM4	101	-9.82	LM10	66	-5.86
LM5	105	-8.79	**LM11**	**1,521**	**-16.48**
LM6	84	-7.82			

**Table 8:** Analysis of the LMs generated for the TCL ligand.

LM	Total of instances	Average FEB (kcal/mol)	LM	Total of instances	Average FEB (kcal/mol)
**LM1**	**522**	**-9.03**	LM13	27	-8.63
**LM2**	**49**	**-8.94**	LM14	30	-8.45
**LM3**	**145**	**-8.97**	LM15	17	-8.53
LM4	24	-8.81	LM16	78	-8.66
**LM5**	**927**	**-8.90**	**LM17**	**88**	**-9.08**
LM6	162	-8.84	LM18	315	-8.86
LM7	34	-8.76	**LM19**	**49**	**-8.89**
LM8	29	-8.72	LM20	107	-8.71
LM9	44	-8.64	LM21	27	-8.78
LM10	58	-8.82	LM22	49	-8.54
LM11	37	-8.52	LM23	2	-4.96
LM12	17	-8.68			

**Table 9:** Analysis of the LMs generated for the ETH ligand.

LM	Total of instances	Average FEB (kcal/mol)	LM	Total of instances	Average FEB (kcal/mol)
LM1	1,263	-6.71	LM11	6	-6.18
LM2	517	-6.62	LM12	17	-6.39
LM3	48	-6.65	**LM13**	**321**	**-7.18**
LM4	47	-6.52	**LM14**	**243**	**-7.03**
LM5	12	-6.47	**LM15**	**43**	**-6.97**
LM6	6	-6.26	**LM16**	**137**	**-7.01**
LM7	5	-6.21	**LM17**	**137**	**-6.93**
LM8	14	-6.48	**LM18**	**21**	**-6.80**
LM9	2	-6.35	LM19	177	-6.75
LM10	27	-6.56			

## Discussion

To verify which snapshots were selected and whether these were the best ones we carefully evaluated their related FEB values. In doing so we organized the instances of each test set according to the FEB in an ascending order (FEB list). Then, we investigated if the selected snapshots were at the top of this list (consequently, with the most negative FEB values). As a result we obtained the data described in Table 10. In column 1 we have the ligand names; in columns 2, 3 and 4 the total number of selected snapshots that are in the top 10 FEB list, top 100 FEB list and top 1,000 FEB list, respectively. Column 5 shows the total number of selected snapshots and the total number of snapshots for each ligand test set.

**Table 10:** Results of the analyzes of the LMs for all four ligands in this study.

Ligand	Top 10 FEB list	Top 100 FEB list	Top 1,000 FEB list	Total selected snapshots/Total snapshots
NADH	10	100	998	1,521/2,823
TCL	10	100	610	1,780/2,737
PIF	10	100	1,000	2,085/3,042
ETH	10	92	617	902/3,043

Based on the data described in Table 10 we can notice that our snapshot selection strategy worked well for all four ligands. For NADH and PIF, from the 10, 100 and 1,000 best FEBs, our method worked best, selecting almost 100% of the best snapshots. For ETH, our method selected the 10 best ones, 92% of the 100 best ones and 617 of the 1,000 best ones. However, for this ligand the method selected less snapshots (617 out of 902). Nonetheless this represents almost 70% of the 1,000 best FEBs. The worst results were obtained for TCL. Only 60% of the top 1,000 FEB list were selected.

In this paper our main contribution is the snapshot selection strategy which was capable of picking up the most promising receptor snapshots based on their conformations. Even if some of the selected snapshots did not have the best FEB values, it is important to test them for they have may have a promising conformation. Besides the analysis about the most promising snapshots, the model trees can indicate the most important residues to predict the good and bad values of FEB. With the results described in this paper we are able to discuss, for instance, about the residue THR100 of NADH model tree (Figure 5). As we show in the results section, for this ligand our methodology selected just one LM, the LM11 (Table 7). If you observe in the model tree, all the snapshots in which the distance of the residue THR100 to the NADH ligand is bigger than 11.49 Å are considered promising snapshots. The discussion about the consequences of this result is beyond the scope of this work but it is a important particularity that must be investigated by a domain specialist and is part of our future work.

## Conclusions

Molecular docking experiments that consider fully flexible-receptor involve typically several types of data describing receptor and ligand conformations and generate a huge amount of data. We analysed docking experiments of the highly flexible InhA enzyme receptor from *M. tuberculosis* with four distinct ligands: NADH, PIF, TCL, and ETH. These experiments were conceived for snapshots obtained from a 3,100 ps MD simulation trajectory. Currently, we have a total of 100,504 valid docking results, 12,424,800 receptor coordinates, and 2,223,098 ligand coordinates. Having these docking results, our goal was to propose a methodology to knowledge discovery, that is, to select the most promising receptor conformations, by identifying snapshots characteristics for their selection. To achieve this goal we used the M5P model tree algorithm for data mining, aiming at identifying representative LM for snapshot selection.

We systematically preprocessed our molecular docking results, mined, and further post processed them to select a set of the most promising receptor conformations from the initial 3,100 snapshots. Preprocessing was done by calculating the shortest interatomic distance between the ligand and the receptor’s residues, for each docking result. They were the predictive attributes. The target attribute was the FEB value. We proposed a strategy to select LMs that can represent the most promising snapshots: the ones to be selected. Our results showed that the inferred model trees were able to select snapshots properly with high correlation values, except for ETH. As future work we intend to further the use of the most promising selected snapshots to perform virtual screening in small molecule libraries using a reduced set of snapshots, that still represents a fully-flexible model of the InhA protein receptor, thus reducing the time to find new druggable compounds candidates for new treatment against tuberculosis.

## Competing interests statement

The authors declare that they have no competing interests.

## Authors' contributions

KSM and ATW executed the preprocessing for the data mining experiments, performed all the data mining experiments and post processing, evaluated the models results and wrote the first draft of the article. DDAR helped to conceive the test cases, to generate different data mining inputs, to perform the data mining experiments and to evaluate the models. ONS helped to define the problem, to analyze the results and to write the final version of the article. All authors read and approved the final manuscript.
